# Human capital and lifetime income gains of scaling-up small-quantity lipid nutrient supplements among children under 2 years: A modelling analysis

**DOI:** 10.1371/journal.pgph.0004388

**Published:** 2025-04-16

**Authors:** Nandita Perumal, Goodarz Danaei, Günther Fink, Mark Lambiris, Christopher R. Sudfeld

**Affiliations:** 1 Department of Epidemiology and Biostatistics, Arnold School of Public Health, University of South Carolina, Columbia, South Carolina, United States of America; 2 Department of Global Health and Population, Harvard TH Chan School of Public Health, Boston, Massachusetts, United States of America; 3 Department of Epidemiology, Harvard T.H. Chan School of Public Health, Boston, Massachusetts, United States of America; 4 Department of Epidemiology and Public Health, Swiss Tropical and Public Health Institute, Basel, Switzerland; 5 Health Economics Facility, Faculty of Medicine, University of Basel, Basel, Switzerland; 6 Department of Nutrition, Harvard TH Chan School of Public Health, Boston, Massachusetts, United States of America; African Population and Health Research Center, KENYA

## Abstract

Undernutrition in early childhood is associated with adverse health and developmental outcomes later in life and remains a persistent global public health problem. Providing small-quantity lipid nutrient supplements (SQ-LNS) to children aged 6-24 months improves child growth and neurodevelopmental outcomes, but the potential long-term benefits to human capital have not been previously estimated. We estimated the potential returns to schooling and lifetime income attributable to increasing coverage of SQ-LNS for children <2 years of age from 0% to 50% or 90% per five-year birth cohort in five countries (Bangladesh, Burkina Faso, Ethiopia, Pakistan, and Uganda) with a high burden of undernutrition. Random-effects meta-analyses were used to estimate the effect of SQ-LNS on child development using evidence from randomized controlled trials, and to estimate the returns to lifetime income as a function of change in development based on a *de novo* meta-analysis of observational economic studies. Gains in school years attributable to scaling-up SQ-LNS to 90% coverage ranged from 0.14 million school years (95% uncertainty interval [UI]: 0.064, 0.25) in Burkina Faso to 1.18 million school years (95%UI: 0.54, 2.11) in Pakistan per five-year birth cohort. With an effect size of 18% return in income per one standard deviation increase in development, the estimated gains in lifetime income ranged from $US 0.41 billion (95% UI: 0.20, 0.68) in Burkina Faso to $US 6.91 billion (95% UI: 3.32, 11.4) in Pakistan per five-year birth cohort. Returns in income per child were above the estimated per child cost of providing SQ-LNS. These findings demonstrate that scaling-up SQ-LNS among children aged 6-24 months may lead to substantial human capital gains in countries with a high-burden of child undernutrition. Longitudinal studies on the long-term effects of SQ-LNS are needed to refine model parameters and to better characterize the impacts on broader health and human capital outcomes.

## Introduction

Globally, an estimated 249 million children under the age of 5 years do not meet their full developmental potential, with countries in sub-Saharan Africa and South Asia bearing a disproportionate burden [1,2]. Optimal nutrition during pregnancy and in early childhood is a central component of the nurturing care framework to support optimal child neurodevelopment [3]. Adequate availability of nutritious foods early in life is essential for neurodevelopmental processes that occur most rapidly during this time [4]. Young children 6-24 months of age are particularly vulnerable to nutritional deficiencies during the period of transition from exclusive or predominant breastfeeding to complementary feeding given that high-density and high-quality foods are required to meet the nutrient demands of the growing infant [5]. Many nutrition interventions, including micronutrient powders, which can be added to home-based complementary foods to enhance the dietary quality of foods consumed by the infant [6], and nutrition education provided to the caregiver to encourage optimal feeding practices [7], have been previously evaluated with the aim of improving nutritional status among young children [7]. While such interventions have shown to reduce the risk of anemia and iron deficiency and have modest benefits for improving growth in early childhood, small-quantity lipid nutrient supplements (SQ-LNS) are one of the very few interventions to have demonstrated benefits for both early childhood growth and neurodevelopmental outcomes [7,8].

SQ-LNS are a nutrient dense paste provided in small-packets that can be added to foods or are consumed as a snack to improve the nutrition quality and quantity of complementary foods provided to young children [9]. Evidence from individual-level participant data meta-analyses from 14 randomized controlled trials conducted in low and middle-income countries (LMICs), with a sample size of greater than 30,000 infants, have shown that young children 6 to 24 months of age who received daily SQ-LNS, compared to standard of care, had improved growth and neurodevelopmental outcome scores, including language, motor, and socio-emotional development scores [10–12]. While previous studies have evaluated the effectiveness and cost-effectiveness of scaling up SQ-LNS based on reductions in child mortality, undernutrition, and disability-adjusted life years [13], SQ-LNS may also confer benefits to longer term human capital outcomes, which have not previously been estimated. We modeled the potential gains in years of schooling and lifetime earnings of scaling-up SQ-LNS from 0 to 50% or 90% coverage among children 6-24 months in five countries with a high-burden of undernutrition where provision of SQ-LNS at the population-level is likely to improve growth and developmental outcomes of all children.

## Materials and methods

**Fig 1:**
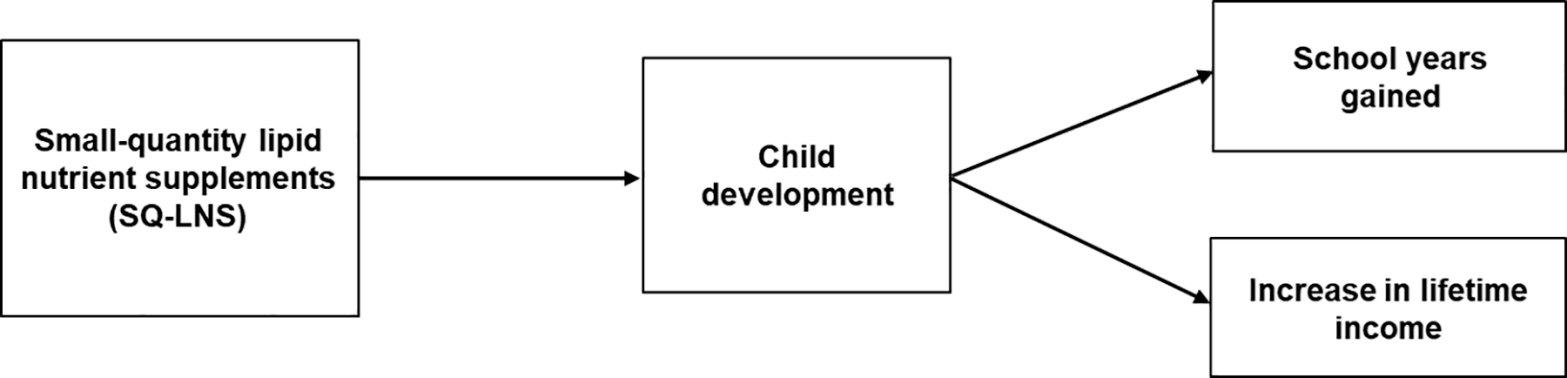
Conceptual framework used to model the potential impact of scaling up SQ-LNS for children between 6-24 months on human capital and income gains.

We used population-based linear deterministic mathematical models to estimate the human capital benefits for scaling up SQ-LNS by predicting two quantities: (1) the increase in school years due to potential gains in development, and (2) the increase in lifetime income due to gains in development (Fig 1). Linear deterministic models quantify the average behavior of a population given a set of parameter values assuming that the system does not dynamically change with downstream consequences [14]. To ‘link’ each component of the model, we used pooled effect sizes from randomized controlled trials to quantify the effect of SQ-LNS on child development (linking the first and second components of the model), from longitudinal birth cohort studies to quantify the gains in years of schooling as a function of higher child development (linking child development to schooling), and from studies that estimated the economic returns attributable to cognitive ability (linking child development to lifetime income).

We estimated the potential gains of scaling up SQ-LNS from current coverage level, which is assumed to be 0%, to two target coverage of 50% and 90% in the five countries – Bangladesh, Burkina Faso, Ethiopia, Pakistan, and Uganda. These countries were selected as exemplars in both Sub-Saharan Africa and South Asia that have a high burden of undernutrition in young children (prevalence of stunting [height-for-age z-score <-2 standard deviation] ranging from 24% to 34% and prevalence of wasting [weight-for-height z-score <-2 standard deviation] ranging from 3.6% to 11% [15]), and a simultaneously high risk of poor development in young children independent of stunting prevalence and poverty [1,16]. Importantly, in countries with a high burden of undernutrition, scaling up SQ-LNS as a population-level strategy for all children 6-24 months of age is likely to maximize the returns on investment as all children are expected to benefit. We selected 50% target coverage levels as an ‘interim’ scenario to model intervention benefits if half of the population of children <2 years of age received the intervention, and 90% target coverage to model an ‘ideal’ scenario in which almost all children <2 years of age living in countries with a high burden of malnutrition received the intervention.

### Quantifying the effect of SQ-LNS on child development

**Fig 2:**
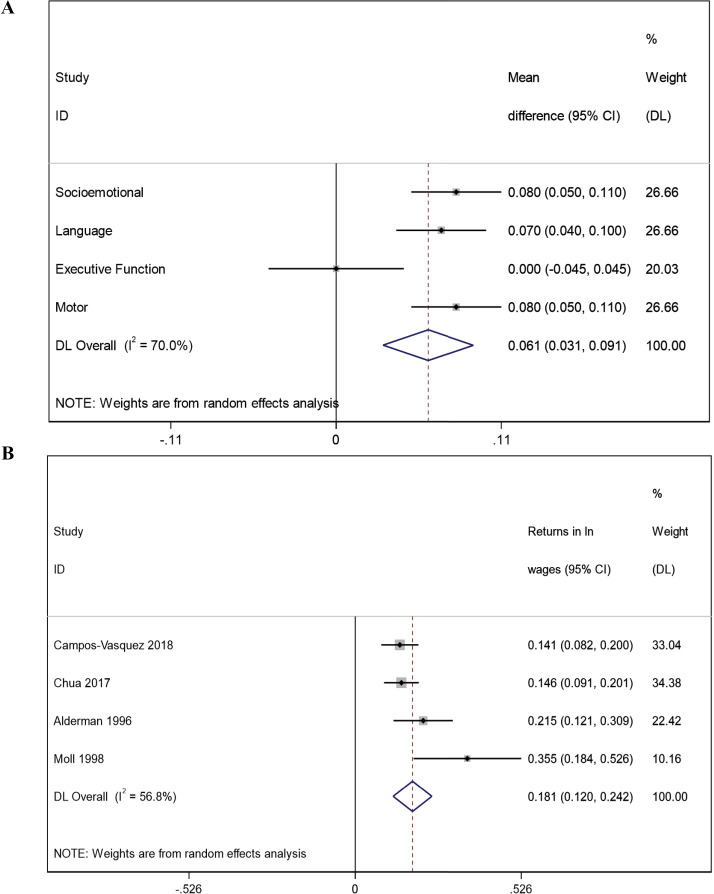
Forest plots of random-effects meta-analyses estimating the pooled effect of: (i) small-quantity lipid nutrient supplement (SQ-LNS) on child development (Panel A); and (ii) returns to income for one standard deviation increase in development (Panel B).

To quantify the effect of SQ-LNS on child development, we used domain-specific pooled estimates from a previously conducted meta-analysis of individual randomized controlled trials (n=13) that evaluated the effect of SQ-LNS on the child development [11]. Briefly, the meta-analysis combined trial specific estimates for each domain of development (motor: n=12 trials, 23899 children; language: n=13 trials, 24561 children; socio-emotional: n = 11 trials, 23588 children; and executive function: n=7 trials, 9095 children) using inverse variance weighting and robust standard errors for cluster-randomized trials as appropriate [11]. For this study, we further pooled the four domain-specific language, socioemotional, executive function, and motor development z-scores estimates from the Prado et al. meta-analysis to quantify the effect of providing SQ-LNS on child development overall (Fig 2A). We included all individual domains of development in the random-effects meta-analysis, irrespective of statistical significance, to be conservative and inclusive in our approach. The overall pooled effect of SQ-LNS was 0.061 SD mean difference in development among young children who received SQ-LNS compared to standard of care (Fig 2A).

### Quantifying the effect of changes in child development on schooling and lifetime income

There is limited longitudinal evidence from LMICs on the direct effect of providing SQ-LNS in early childhood on human capital outcomes, such as schooling and lifetime income, given the substantial resources required for following trial participants throughout the life course. As a result, we used longitudinal data from robust observational cohorts in LMICs to estimate the change in number of years of schooling and lifetime income that may be anticipated with improvements in child development in early life. To estimate the potential gains in years of schooling as a function of higher child development, we used estimates from the COHORTS collaboration, which includes data from population-based birth cohorts in six LMICs [17]. One standard deviation change in child development assessed at 4.0-8.5 years of age was associated with an average 0.82 years (95% CI: 0.47, 1.16) additional years of schooling attained [17]. To estimate the effect of early childhood development on gains in lifetime income, we evaluated individual studies that were included in two systematic reviews that estimated the economic returns attributable to cognitive ability [18,19] for the following eligibility criteria: (i) primary data from participants in LMICs; and (ii) the study did not adjust for mediators, such as schooling, when estimating the relationship between early childhood development and returns to income. We restricted our analysis to only studies conducted in LMICs and those that did not over-adjust for important mediators to ensure generalizability of the estimates. Only four studies (conducted in Pakistan, South Africa, Mexico, and a multi-country survey of 12 LMICs) met these inclusion criteria and were included in the meta-analysis for this study [20–23]. Using a random effects meta-analysis of four studies assessing the effect of early developmental outcomes on returns to wages showed that one standard deviation increase in cognitive ability was associated with an 18.1% increase (95%CI: 12.0, 24.2) in lifetime income on average (Fig 2B).

### Quantifying the net present value of future income

For estimating gains in adult income, the net present value of lifetime income was estimated by summing the country-specific discounted annual income over a 40-year working period (i.e., 20 to 59 years), assuming an annual income to be 2/3 of per capita gross domestic product in each country extracted from the World Development Indicators [24,25]. This approach has been previously used when wage data are not easily accessible [26]. We calculated the country-specific sum of discounted lifetime income in 2015 constant US$ based on a 3% discounting rate, assuming an annual real wage growth of 2%, and country-specific survival probabilities for each working year from 20 to 59 years. The gross domestic product in 2015 constant US dollars was used to ensure comparability of benefit estimates across countries which can be directly compared with costs of the interventions. However, we also estimated the lifetime income benefits adjusted for purchasing power (reference year 2017) to account for the variation in the relative local value of income.

### Quantifying population-level human capital benefits

Once we identified estimates to ‘link’ each component of the model, we derived population-level changes in years of schooling and lifetime income by multiplying the effect size of the estimated change in development due to SQ-LNS supplementation by the effect sizes for: (i) gains in school years, and (ii) returns to income, respectively, and scaled these estimates to the 2015-2020 five-year birth cohort size of each country. The birth cohort size accounted for the probability of survival up to 6 months of age as only the proportion of children alive at 6 months of age would be eligible to receive the SQ-LNS intervention. Data on the five-year birth cohort size and the probability of survival up to 6 months was retrieved from the United Nations World Population Prospects 2020 Revision [27]. To understand what these total economic benefits would mean at the individual level, we further estimated the country-specific returns in lifetime income per child by dividing the total income gains per birth cohort by the number of children who would be targeted to receive SQ-LNS. The 95% uncertainty intervals around the final estimates were calculated by using the 2.5^th^ and 97.5^th^ percentiles of 1000 simulations. All estimates were generated using Stata 16 Statistical Software package (StataCorp LP).

## Results

Across the five countries with a high burden of undernutrition, scaling-up SQ-LNS to 90% coverage was estimated to yield substantial gains in years of schooling and lifetime income per 5-year birth cohort (Table 1). The largest gains in school years were estimated for Pakistan with 1180 thousand school years per birth cohort (95% UI: 541, 2106) and Bangladesh with 609 thousand school years per birth cohort (95% UI: 279, 1087), followed by estimated gains in Ethiopia, Uganda, and Burkina Faso in descending order. Similarly, the absolute gains in lifetime income per birth cohort (95% UI) was estimated to be $6.91 billion (3.32, 11.4) in Pakistan, $4.06 billion (1.95, 6.72) in Bangladesh, $3.84 billion (1.84, 6.35) in Ethiopia, $1.13 billion (0.54, 1.87) in Uganda, and $0.41 billion (0.20, 0.68) in Burkina Faso. Overall, the largest absolute returns to income were observed for countries with larger population sizes and higher annual wages. Inferences regarding benefits in lifetime income were similar when using international dollars, but the absolute magnitude of the estimated gains were larger when accounting for purchasing

**Table 1:** Impact of scaling up small-quantity lipid nutrient supplements on labour market outcomes, through improvements in development.

Country	Target Coverage (%)	Total school years gains in 1000s (95% UI: LB, UB)	Total income gains in 2015 $US millions (95%UI: LB, UB)	Additional future income per child in 2015 $US (95% UI: LB, UB)
**Bangladesh**	50	339 (155, 604)	2,255 (1082, 3732)	313 (150, 517)
	90	609 (279, 1087)	4,058 (1947, 6717)	
**Burkina Faso**	50	77 (35, 138)	228 (110, 378)	127 (61, 211)
	90	139 (64, 248)	411 (197, 680)	
**Ethiopia**	50	383 (175, 682)	1,280 (614, 2119)	150 (72, 249)
	90	688 (316, 1228)	2,304 (1106, 3814)	
**Pakistan**	50	656 (301, 1170)	3,839 (1842, 6354)	269 (129, 445)
	90	1,180 (541, 2106)	6,910 (3316, 11437)	
**Uganda**	50	174 (80, 310)	626 (300, 1036)	159 (76, 263)
	90	313 (143, 558)	1,127 (541, 1866)	

power parity (Table 2).

**Table 2:** Impact of scaling up small-quantity lipid nutrient supplements on lifetime income in international dollar accounting for purchasing power parity.

Country	Target Coverage (%)	Benefits by cohort: Lifetime income in 2017 International $ millions	Returns in lifetime earnings per child in 2017 International $
**Bangladesh**	50%	6682 (3207, 11060)	926 (444, 1533)
	90%	12028 (5772, 19908)	
**Burkina Faso**	50%	673 (323, 1114)	376 (180, 622)
	90%	1211 (581, 2005)	
**Ethiopia**	50%	3556 (1706, 5886)	418 (200, 691)
	90%	6401 (3071, 10594)	
**Pakistan**	50%	12106 (5809, 20037)	847 (407, 1402)
	90%	21790 (10456, 36066)	
**Uganda**	50%	1528 (733, 2529)	388 (186, 643)
	90%	2751 (1320, 4553)	

There were also large returns in terms of additional future income per child, with largest absolute increase in benefits to income per child estimated to be in Bangladesh ($313 per child, 95% IU: 150, 517), followed by Pakistan ($269 per child, 95%UI: 129, 445), Uganda ($159, 95%UI: 76, 263), Ethiopia ($150, 95%UI: 72, 249), and Burkina Faso ($127, 95%UI: 61, 211) (Table 1). The returns in income per child were greater in Bangladesh and Pakistan due to higher annual wages as compared to Uganda, Ethiopia and Burkina Faso.

## Discussion

The findings of this study suggest that scaling-up SQ-LNS among children 6-24 months of age may lead to substantial population-level gains in educational attainment and lifetime in countries with a high burden of undernutrition. We generated estimates for five countries in Sub-Saharan Africa and South Asia as exemplars for a population-based approach to scaling up SQ-LNS. The potential benefits ranged from 139 thousand additional school years and $US 0.41 billion in lifetime income gains in Burkina Faso to 1.18 million additional school years and $US 6.91 billion in lifetime income gains in Pakistan. At the individual-level, the absolute gains in future income per child ranged from $US 127 in Burkina Faso to $US 313 in Bangladesh, which are greater than the estimated cost per child of providing SQ-LNS through the healthcare system in Burkina Faso (estimated to be $71), rural Uganda (estimated to be $52), and Bangladesh (estimated to be $48), among others [13,28–30].

Diets with adequate quality and quantity are essential for supporting optimal child development in early life. The Copenhagen Consensus recently identified SQ-LNS as being one of the ‘best-bets’ for investing in nutrition intervention due to an average benefit-cost ratio of 13.7 across 40 LMICs attributable to cases of stunting and all-cause mortality averted [31]. Similarly previous frameworks to assess the cost-effectiveness of scaling up SQ-LNS among young children in LMIC have focused on number of cases of stunting, wasting, anemia, developmental disability, and/or all-cause mortality averted [30]; finding that SQ-LNS is a highly cost-effective intervention based on the number of life years saved and the number of disability-adjusted life years averted. If these analyses of cost-effectiveness and benefit-cost ratios were to further incorporate the estimated benefits for human capital outcomes, including educational attainment, SQ-LNS would be considered even more cost effective as an intervention. Indeed, the returns to future income per child for scaling up SQ-LNS are higher than the estimated returns from scaling up other nutrition interventions during pregnancy, such as multiple micronutrient supplements, at the population level [26]. The product cost of SQ-LNS is ∼$0.061 per 20-g sachet based on the cost-effectiveness evaluation of a program to provide daily provision of SQ-LNS to all children aged 6-18 months in rural Uganda through village health teams [13]. Of the estimated per child cost of $52 for providing SQ-LNS in this context, approximately $22 of the total cost per child was the cost of the product (equivalent to ~43% proportionally), ∼$11 was attributable to international shipping and handling, customs clearance and domestic transport, storage and handling, and ∼$19 was non-product programmatic costs [13]. The implementation costs associated with scaling up SQ-LNS may be lower (e.g., in Burkina Faso [28,30]) depending on adaptability and reach of existing healthcare systems and programs.

It is important to note that we estimated benefits in lifetime income and educational attainment of scaling up SQ-LNS based on improvement in child development alone, and therefore, potentially underestimated the overall impact that could be accrued through other benefits of SQ-LNS, such as improvements in early childhood growth, hemoglobin concentration, and overall health (e.g., fewer illness episodes). In countries where SQ-LNS might be coupled with interventions that specifically target developmental outcomes, including responsive care programs that have a greater impact on child development outcomes [3], the returns on investment are likely to be even higher. Although we estimated the pooled effect size for child development using meta-analyzed estimates of developmental domains published by Prado and colleagues [11], we did not use multi-level meta-analyses using original trial-specific estimates to account for nesting of domains within trials, which may affect the variance estimation of the pooled effect size. Nonetheless, we used a conservative approach by using the pooled mean differences in developmental scores from all trials of SQ-LNS, which were conducted in settings that may not have had as severe a burden of undernutrition as in the exemplar countries selected for this study. For example, in the individual participant data analysis led by Prado and colleagues [11], the effect of SQ-LNS was greater in subgroup of study sites (Bangladesh, Burkina Faso, Madagascar, Mali, Malawi and Zimbabwe) with a higher prevalence of stunting at 18-months of age for the study participants compared to the control group – effect sizes ranged from 0.11-0.13 mean SD difference for language, socio-emotional and motor development (though not executive function). Although there were substantial estimated benefits of scaling-up SQ-LNS on schooling and lifetime income, there were large uncertainties in these estimates driven largely by the uncertainty in estimates of school years gained and returns to income associated with increases in cognition. For example, given the paucity of longitudinal evidence between child development <2 years of age and human capital outcomes, we used an estimate of the association between developmental scores and years of schooling that may differ based on age at which development was assessed. More studies are needed to refine model parameters and better characterize the effects of SQ-LNS interventions on human capital outcomes using data from long-term follow-up of participants in intervention studies. Lastly, we used a linear population-averaged deterministic model that has the advantage of quantifying the cause-and-effect relationships efficiently and provide clear interpretation of the model estimates, particularly in the context of sparse data on implementation factors that vary between countries. We did not, however, incorporate modelling uncertainty due to stochastic factors in complex systems, which are not easily quantifiable, and as such, our uncertainty intervals may be considered as a lower bound. Nonetheless, we incorporated evidence from the most recent, robust evidence from randomized controlled trials or longitudinal cohort studies and used uncertainty propagation methods to model uncertainties in all parameters to account for heterogeneity of SQ-LNS effects in various contexts.

Overall, this study provides further evidence to support initiatives to advance the implementation and scaling-up of SQ-LNS among young children in populations with a high prevalence of child undernutrition. While substantial evidence now exists to demonstrate the efficacy of SQ-LNS, further research is needed to understand the costs and impact of context-specific implementation strategies. Programs and approaches that target the most vulnerable populations where young children are disproportionately affected by biological or sociodemographic vulnerabilities are likely to yield the largest absolute human capital benefits of scaling up SQ-LNS.
